# Microbiability of meat quality and carcass composition traits in swine

**DOI:** 10.1111/jbg.12504

**Published:** 2020-09-26

**Authors:** Piush Khanal, Christian Maltecca, Clint Schwab, Justin Fix, Francesco Tiezzi

**Affiliations:** ^1^ Department of Animal Science North Carolina State University Raleigh NC USA; ^2^ The Maschhoffs LLC Carlyle IL USA

**Keywords:** heritability, meat quality and carcass composition traits, microbiability, microbial diversity, microbiome

## Abstract

The impact of gut microbiome composition was investigated at different stages of production (weaning, Mid‐test and Off‐test) on meat quality and carcass composition traits of 1,123 three‐way crossbred pigs. Data were analysed using linear mixed models which included the fixed effects of dam line, contemporary group and gender as well as the random effects of pen, animal and microbiome information at different stages. The contribution of the microbiome to all traits was prominent although it varied over time, increasing from weaning to Off‐test for most traits. Microbiability estimates of carcass composition traits were greater than that of meat quality traits. Among all of the traits analysed, belly weight (BEL) had a higher microbiability estimate (0.29 ± 0.04). Adding microbiome information did not affect the estimates of genomic heritability of meat quality traits but affected the estimates of carcass composition traits. Fat depth had a greater decrease (10%) in genomic heritability at Off‐test. High microbial correlations were found among different traits, particularly with traits related to fat deposition with a decrease in the genomic correlation up to 20% for loin weight and BEL. This suggested that genomic correlation was partially contributed by genetic similarity of microbiome composition. The results indicated that better understanding of microbial composition could aid the improvement of complex traits, particularly the carcass composition traits in swine by inclusion of microbiome information in the genetic evaluation process.

## 1 INTRODUCTION

The mammalian gastrointestinal tract is a home of a diverse microbiota population which serve various biological functions of the host (Frese, Parker, Calvert, & Mills, 2015). Gut microbiota have recently been the target of many research efforts resulting from the rapid development in molecular technologies and led to a vast influx of “omics” studies (Guevarra et al., 2019). The importance of gut microbiota is widely accepted (Kim et al., 2011), with commensal bacteria often being called the “forgotten organ” of the host (O’Hara & Shanahan, 2006), impacting hosts in a multitude of ways. For example, the microbial composition helps in promoting the gastrointestinal health through metabolites, postnatal development, degradation of short‐chain fatty acids and stimulation of immune system (Mann et al., 2014; Pedersen, Andersen, Hermann‐bank, Stagsted, & Boye, 2013; Stappenbeck & Virgin, 2016).

The gut microbiome constitutes a portion of the hologenome (Sommer & Bäckhed, 2013; Xiao et al., 2016) and has the potential to affect numerous biological activities that the hosts lack (Pajarillo, Chae, Balolong, Kim, & Kang, 2014). The microbial diversity of the intestine accounted for a significant amount of the phenotypic variation for any trait in humans and should be accounted for when assessing the heritability not only in human but also in plants and livestock (Sandoval‐Motta, Aldana, Martínez‐Romero, & Frank, 2017). In livestock, Difford, Lassen, and Løvendahl (2016) termed “microbiability” as the proportion of total variance explained by microbiome for performance traits of dairy cattle. Difford et al. (2018) reported the effect of microbiota variation in methane production in dairy cows. Similarly, Ramayo‐Caldas et al. (2020) and Saborío‐Montero et al. (2020) also reported the significant microbiability of methane production in dairy cattle. In pigs, Camarinha‐Silva et al. (2017) and Weishaar, Wellmann, Camarinha‐Silva, Rodehutscord, and Bennewitz (2020) reported the presence of a significant microbiability of daily gain, feed intake and feed conversion rate. The gut microbiome also has a significant impact on porcine fatness (He et al., 2016). In chicken, Wen et al. (2019) demonstrated the significant contribution of caecal and duodenal microbiota in fat deposition. Until recently, selection of different traits in pigs has been done with the use of pedigree and genomic information, yet the advantage of incorporating microbial information in the genetic evaluation processes has not been assessed. Few studies have described the relationship of microbial diversity and host (Guevarra et al., 2019; McCormack et al., 2018) however, these were mostly from a nutritional perspective. Camarinha‐Silva et al. (2017) and Difford et al. (2018) reported the possibility of incorporating host genome and microbiome information for better prediction of phenotypic traits.

Specifically, the contribution of microbial composition to the phenotypic variation of meat quality and carcass composition traits in pigs has yet to be explored and no studies to date have been conducted on the effect of microbial composition at different stages of production on growth and carcass composition. Therefore, the objectives of this study are to estimate the microbiability estimates for different meat quality and carcass composition traits; to investigate the impact of intestinal microbiome on heritability estimates; to estimate the correlation between microbial diversity and meat quality and carcass composition traits; and to estimate the microbial correlation between the meat quality and carcass composition traits in a commercial swine population.

## 2 MATERIAL AND METHODS

Phenotypic records presented in this study came from a commercial farm operated by The Maschhoffs LLC. All methods and procedures were in accordance to the Animal Care and Use policies of North Carolina State University and the National Pork Board. The experimental protocol for faecal sample collection received approval number 15027 from Institutional Animal Care and Use Committee. All pigs were harvested in commercial facilities under the supervision of USDA Food Safety and Inspection Service.

### 2.1 Animals and sample collection

Data were collected from crossbred individuals that were obtained from 28 founding Duroc sires and 747 commercial F_1_ sows composed of Yorkshire × Landrace or Landrace × Yorkshire. The pigs were weaned at 18.64 ± 1.09 days old and were moved to nursery‐finishing facility. Pigs were kept in 334 single‐sire single‐sex pens with 20 pigs per pen. The experiment was repeated six times, each of which comprised of two pens (one pen of female pig and one pen of castrated male). Pigs that came together in one replicate were put together in one contemporary group. The test period began the day that pigs were moved to the nursery‐finishing facility. During the nursery, growth and finishing period all pigs were fed a standard pelleted feed based on sex and live weight. Feed and water were provided ad libitum to pigs. Details of diet and their nutritional values are provided in Table S1. The pigs received a standard vaccination and medication routine (Tables S2–S4). End of test (Off‐test) was reached when the average weight of pigs of each pen reached 138 kg. The average age at Off‐test was 196.4 ± 7.80 days. Faecal samples for 16S rRNA sequencing were collected as follows. Rectal swabs were collected from all pigs at three stages from respective pens: weaning (Wean), 15 weeks postweaning (Mid‐test; average 118.2 ± 1.18 days) and Off‐test. Four to five pigs from each pen were selected as detailed by (Wilson et al., 2016). The selected pigs per pen represented an average pig for body weight, along with pigs approximately 1 and 2 *SD* above and below the pen average. Their rectal swabs were used for subsequent microbial sequencing. There were 1,205, 1,295 and 1,273 samples at weaning, Mid‐test and Off‐test, respectively. Distribution of samples across families, time points and sex are provided (Table S5).

### 2.2 Illumina amplicon sequencing

DNA extraction, purification, Illumina library preparation and sequencing were done as described by (Lu et al., 2018). Briefly, total DNA (gDNA) was extracted from each rectal swab by mechanical disruption in phenol: chloroform: isoamyl alcohol solution. Bead‐beating was performed on the Mini‐BeadBeater‐96 (MBB‐96; BioSpec) for 4 min at room temperature, and samples were centrifuged at 3,220 *g*. The DNA was then purified using a QIAquick 96 PCR purification kit (Qiagen). Minor modifications were performed in purification process of manufacturer's instruction. The modification included the addition of sodium acetate (3 M, pH 5.5) to Buffer PM to a final concentration of 185 mM, combination of crude DNA with 4 volumes of Buffer PM and elution of DNA in 100 µL Buffer EB. All sequencing was performed at DNA Sequencing Innovation Laboratory at the Center of Genome Sciences and Systems Biology at Washington University in St. Louis. Phased, bi‐directional amplification of the v4 region (515–806) of the 16S rRNA gene was employed to generate indexed libraries for Illumina sequencing as described in Faith et al. (2013). Sequencing was performed on an Illumina MiSeq instrument (Illumina, Inc.), generating 250‐bp paired‐end reads.

### 2.3 16S rRNA gene sequencing and quality control of data

Sequencing of 16S rRNA gene and quality control of data were done as described by Lu et al. (2018). Briefly, the pairs of 16S rRNA gene sequences obtained from Illumina sequencing were combined into a single sequence using FLASH v1.2.11 (Magoc & Salzberg, 2011). The sequences with a mean quality score below Q35 were filtered out using PRINSEQ v0.20.4 (Schmieder & Edwards, 2011). Then, the forward‐oriented sequences were matched with primer sequences and trimmed off. Mismatch was allowed up to 1 base pair. Sequences were subsequently demultiplexed using QIIME v1.9 (Caporaso et al., 2010). QIIME was used to cluster the nucleotide sequences into operational taxonomic units (OTU) as explained by Lu et al. (2018). A modified version of Greengenes (Ley, Turnbaugh, Klein, & Gordon, 2006; Schloss & Handelsman, 2006) was used as reference database. Ninety per cent of the input sequences were matched to the reference database. The remaining 10% which did not match to the reference database were then clustered de novo with UCLUST (Schloss & Handelsman, 2006) to generate new reference OTU. Then, the 90% of reads that were matched with the reference database were again assigned to the new reference OTU that were derived from the de novo cluster. The sparse OTU were filtered to get a minimum total observation count of 1,200 (0.05% of the samples) to be retained. This threshold, albeit arbitrary, was set in order to discard the features that were considered unreliable in the OTU generation process. The resulting OTU table was rarefied to 10,000 counts per sample, and 1,755 OTU were retained for further analysis.

### 2.4 Genotyping

All pigs were genotyped with the PorcineSNP60 v2 BeadChip (Illumina, Inc.). Quality control procedures were applied by removing the SNPs that had call rate <0.90 and minor allele frequency <0.05. After quality control, the number of SNPs remaining for further analyses was 42,529.

### 2.5 Phenotypic data

Phenotypic data collection was done as described by (Wilson et al., 2016). Meat quality traits (intramuscular fat content [IMF], Minolta a* [MINA], Minolta b* [MINB], Minolta L* [MINL], ultimate pH [PH], subjective colour score [SCOL], subjective marbling score [SMARB], subjective firmness score [SFIRM], shearing force [SSF]) and carcass composition traits (belly weight [BEL], ham weight [HAM], loin weight [LOIN], fat depth [FD], loin depth [LD] and carcass average daily gain [CADG]) were used for the current analysis. All the traits were measured as described by Khanal, Maltecca, Schwab, Gray, and Tiezzi (2019). A summary of traits used in current analysis is reported in Table 1.

**TABLE 1:** Descriptive statistics of carcass composition and meat quality traits: acronym, mean, *SD* and coefficient of variation (CV) values

Traits	Acronym	Mean	*SD*	CV, %
Carcass composition traits				
Loin depth, mm	LD	67.99	7.21	10.60
Back fat depth, mm	FD	22.07	5.24	23.74
Carcass average daily gain, g/day	CADG	552.90	73.93	13.73
Ham weight, kg	HAM	25.19	2.34	9.29
Loin weight, kg	LOIN	20.01	1.88	9.39
Belly weight, kg	BEL	15.88	2.55	16.06
Meat quality				
Intramuscular fat, %	IMF	2.71	1.01	37.26
Minolta a*	MINA	3.77	1.16	30.76
Minolta b*	MINB	−0.16	0.87	−543.75^a^
Minolta L*	MINL	45.37	5.76	12.70
Ultimate pH	PH	5.64	0.22	3.90
Subjective colour	SCOL	2.72	0.57	20.96
Subjective marbling	SMARB	3.10	0.91	29.35
Subjective firmness	SFIRM	3.05	1.04	34.09
Slice shear force, *N*	SSF	156.96	41.99	2.75

^a^ The value is negative because the value of Minolta b* score ranges from negative to positive value and has mean of negative value.

### 2.6 Statistical analysis

The data were analysed using ASReml v4 (Gilmour, Gogel, Cullis, Welham, & Thompson, 2014). Univariate analyses were conducted to estimate heritability and microbiability estimates, and variance components for each trait. Single trait models were fitted as follows:

(1) $$y_{\mathrm{ijklmn}}\;=\;\mu \;+\;{\text{dl}}_{i}\;+\;{\text{cg}}_{j}\;+\;{\text{sex}}_{k}\;+\;{\text{animal}}_{l}\;+\;{\text{pen}}_{m\left(j\right)}\;+e_{\mathrm{ijklmn}}$$

where *µ* was the overall mean, dl*_i_* was the *i*th fixed effect of dam line (two levels), cg*_j_* was the *j*th fixed effect of the contemporary group (six levels), sex*_k_* was the *k*th fixed effect of sex (two levels), animal*_l_* was the random animal genetic effect, pen*_m_*_(_ *_j_*_)_ was the random effect of pen nested within contemporary group and *e_ijklmn_* was the random residual. Pen and residuals were assumed normally distributed with mean zero and with variances $I\sigma_{\text{pen}}^{2}$ and $I\sigma_{e}^{2}$, respectively, where **I** was an identity matrix. The random effect of animal was assumed normally distributed with mean 0 and variance $G\sigma_{a}^{2}$, where **G** was a realized genomic relationship matrix obtained according to VanRaden (2008) as follows:

$$G=\frac{\left(M‐P\right)\;{\left(M‐P\right)}^{\prime }}{2\sum_{j\;=\;1}^{m}p_{j}\;\left(1‐p_{j}\right)}$$

where **M** is a matrix of marker alleles with *m* columns (*m* = total number of markers) and *n* rows (*n* = total number of genotyped individuals), and **P** is a matrix containing the frequency of the second allele (*p_j_*), expressed as 2*p_j_*. **M_ij_** was −1 if the genotype of individual *i* for SNP *j* was homozygous for the first allele, 0 if heterozygous, or 1 if the genotype was homozygous for the second allele. Narrow sense heritability estimates were estimated as $h^{2}\;=\;\frac{\sigma_{a}^{2}}{\sigma_{P}^{2}}$, with $\sigma_{P}^{2}\;=\;\sigma_{a}^{2}\;+\;\sigma_{\text{pen}}^{2}\;+\;\sigma_{e}^{2}.$

We added the microbiome information to model (1) in order to estimate the changes in heritability due to the incorporation of microbiome information at each stage of sample collection. Model (2) was then:

(2) $$y_{\mathrm{ijklmno}}\;=\;\mu \;+\;{\text{dl}}_{i}\;+\;{\text{cg}}_{j}\;+\;{\text{sex}}_{k}\;+\;{\text{animal}}_{l}\;+{\text{microbiome}}_{m}\;+{\text{pen}}_{n\;\left(j\right)}\;+\;e_{\mathrm{ijklmno}}$$

where dl, cg, sex, animal, pen and *e* were as previously described and microbiome*_m_* was the random effect of the animal microbiome. The effect of the microbiome was assumed normally distributed with mean 0 and variance $O\sigma_{m}^{2}$ in which **O** was a variance–covariance matrix among individuals and $\sigma_{m}^{2}$ was the microbiome variance. The matrix **O** was created following Ross, Moate, Marett, Cocks, and Hayes (2013). Briefly, **O** was obtained as $O\;=\;\frac{1}{q}\;{\mathrm{XX}}^{T}$, with matrix **X** of dimension of *n × q*, where *n* is the number of animals and *q* is the number of OTU. **X** was constructed from **S**, a matrix of equivalent dimensions *n × q*. Each element of the **S** matrix, S*_ij_*, was the relative abundance of OTU *j* in animal *i* (plus 0.001). The elements of *X* were calculated as follows:

$$X_{\mathrm{ij}}\;=\;\frac{\log\;(S_{\mathrm{ij}})‐\;\overset{}{\bar{\log\;S_{.j}}}}{sd\;(\log\;S_{.j})}$$

where *S*_._ *_j_* was the vector of the *j*th column of **S**. The **O** matrix was created for each stage (Wean, Mid‐test and Off‐test) separately and fitted in each model separately with different **O** matrix. The contribution of the microbiome to the overall variance (microbiability) was calculated as follows: $m^{2}\;=\;\frac{\sigma_{m}^{2}}{\sigma_{P}^{2}}$ (Difford et al., 2016). The total variance $\sigma_{P}^{2}$ was in this case obtained as $\sigma_{P}^{2}\;=\;\sigma_{a}^{2}\;+\;\sigma_{m}^{2}\;+\;\sigma_{\text{pen}}^{2}\;+\;\sigma_{e}^{2}.$ Bivariate analyses were subsequently conducted to estimate genomic and microbial correlations among traits. Bivariate models were of form:

(3) $$\left[\begin{array}{c} y_{1}\\ y_{2} \end{array}\right]\;=\;\left[\begin{array}{cc} X_{1} & 0\\ 0 & X_{2} \end{array}\right]\;\left[\begin{array}{c} b_{1}\\ b_{2} \end{array}\right]\;+\;\left[\begin{array}{cc} Z_{1} & 0\\ 0 & Z_{2} \end{array}\right]\;\left[\begin{array}{c} a_{1}\\ a_{2} \end{array}\right]\;+\;\left[\begin{array}{cc} K_{1} & 0\\ 0 & K_{2} \end{array}\right]\;\left[\begin{array}{c} o_{1}\\ o_{2} \end{array}\right]\;+\;\left[\begin{array}{cc} W_{1} & 0\\ 0 & W_{2} \end{array}\right]\;\left[\begin{array}{c} p_{1}\\ p_{2} \end{array}\right]\;+\;\left[\begin{array}{c} e_{1}\\ e_{2} \end{array}\right]$$

where ***y*_1_** and ***y*_2_** were the vectors of phenotypic measurements for trait 1 and trait 2, respectively; ***X*_1_** and ***X*_2_** were the incidence matrices relating the fixed effects to vector ***y*_1_** and vector ***y*_2_**, respectively; ***b*_1_** and ***b*_2_** were the vector of fixed effect for trait 1 and 2, respectively; ***Z*_1_** and ***Z*_2_** were the incidence matrices relating the phenotypic observations to the vector of random animal effects for trait 1 and 2, respectively; ***a*_1_** and ***a*_2_** were the vectors of random animal effect for trait 1 and 2, respectively; ***K*_1_** and ***K*_2_** were the incidence matrices relating the phenotypic observations to the vector of random microbiome effect for trait 1 and 2, respectively; ***o*_1_** and ***o*_2_** were the vectors of random microbiome effect for trait 1 and 2 respectively; ***W*_1_** and ***W*_2_** were the incidence matrices relating the phenotypic observations to the vector of random pen effects for trait 1 and 2, respectively; ***p*_1_** and ***p*_2_** were the vector of random pen effect for trait 1 and 2, respectively; and ***e*_1_** and ***e*_2_** were the vectors of random residuals for trait 1 and 2, respectively. The fixed effects and random effects were the same as fitted in the univariate analyses.

The additive effects were again assumed normally distributed with means 0 and variance $\text{Var}\;\left[\begin{array}{c} a_{1}\\ a_{2} \end{array}\right]\;=\;C\;⊗\;G$, where $C\;=\;\left[\begin{array}{cc} \sigma_{a1}^{2} & \sigma_{a12}\\ \sigma_{a21} & \sigma_{a2}^{2} \end{array}\right]$. The elements of the covariance matrix **C** were defined as follows: $\sigma_{a1}^{2}$, the genetic variance for trait 1, $\sigma_{a2}^{2}$, the genetic variance for trait 2, and $\sigma_{a12}\;=\;\sigma_{a21}$, the additive genetic covariance between trait 1 and 2. Similar assumptions were made for the microbiome effect for which the covariance structure was assumed $\text{Var}\;\left[\begin{array}{c} o_{1}\\ o_{2} \end{array}\right]\;=\;Q\;⊗\;O$, with $Q\;=\;\left[\begin{array}{cc} \sigma_{m1}^{2} & \sigma_{m12}\\ \sigma_{m21} & \sigma_{m2}^{2} \end{array}\right]$. The elements **Q** were: $\sigma_{m1}^{2}$, the microbiome variance for trait 1, $\sigma_{m2}^{2}$, the microbiome variance for trait 2 and $\sigma_{m12}\;=\;\sigma_{m21}$ , the microbiome covariance between trait 1 and 2. The pen (co)variance structure was $\text{Var}\;\left[\begin{array}{c} p_{1}\\ p_{2} \end{array}\right]\;=\;W\;⊗\;I$, with $W\;=\;\left[\begin{array}{cc} \sigma_{\text{pen1}}^{2} & 0\\ 0 & \sigma_{\text{pen}2}^{2} \end{array}\right]$ and **I** an identity matrix. The **W** matrix elements were: $\sigma_{\text{pen1}}^{2}$ and $\sigma_{\text{pen2}}^{2}$ being the pen variance for traits 1 and 2, respectively. Pen effects were assumed uncorrelated among traits. The residual variance was given by $\text{Var}\;\left[\begin{array}{c} e_{1}\\ e_{2} \end{array}\right]\;=\;R\;⊗\;I$, $R\;=\;\left[\begin{array}{cc} \sigma_{e1}^{2} & \sigma_{e12}^{2}\\ \sigma_{e21}^{2} & \sigma_{e2}^{2} \end{array}\right]$ where and **I** was an identity matrix. The components of **R** were defined as follows: $\sigma_{e1}^{2}$ was the residual variance for trait 1, $\sigma_{e2}^{2}$ was the residual variance for trait 2, and $\sigma_{e21}^{2}\;=\;\sigma_{e12}^{2}$ was the residual covariance among the two traits. Preliminary analyses (data not shown) showed that correlations were not estimable for the traits with estimated microbiome variance <3%. Microbial correlations were therefore estimated among traits for which microbiome explained at least 3% of total phenotypic variance. In all cases, microbial correlations were not estimated at weaning since microbiome accounted for <3% of total variance for all traits.

## 3 RESULTS AND DISCUSSION

### 3.1 Phenotypic data summary

**FIGURE 1:**
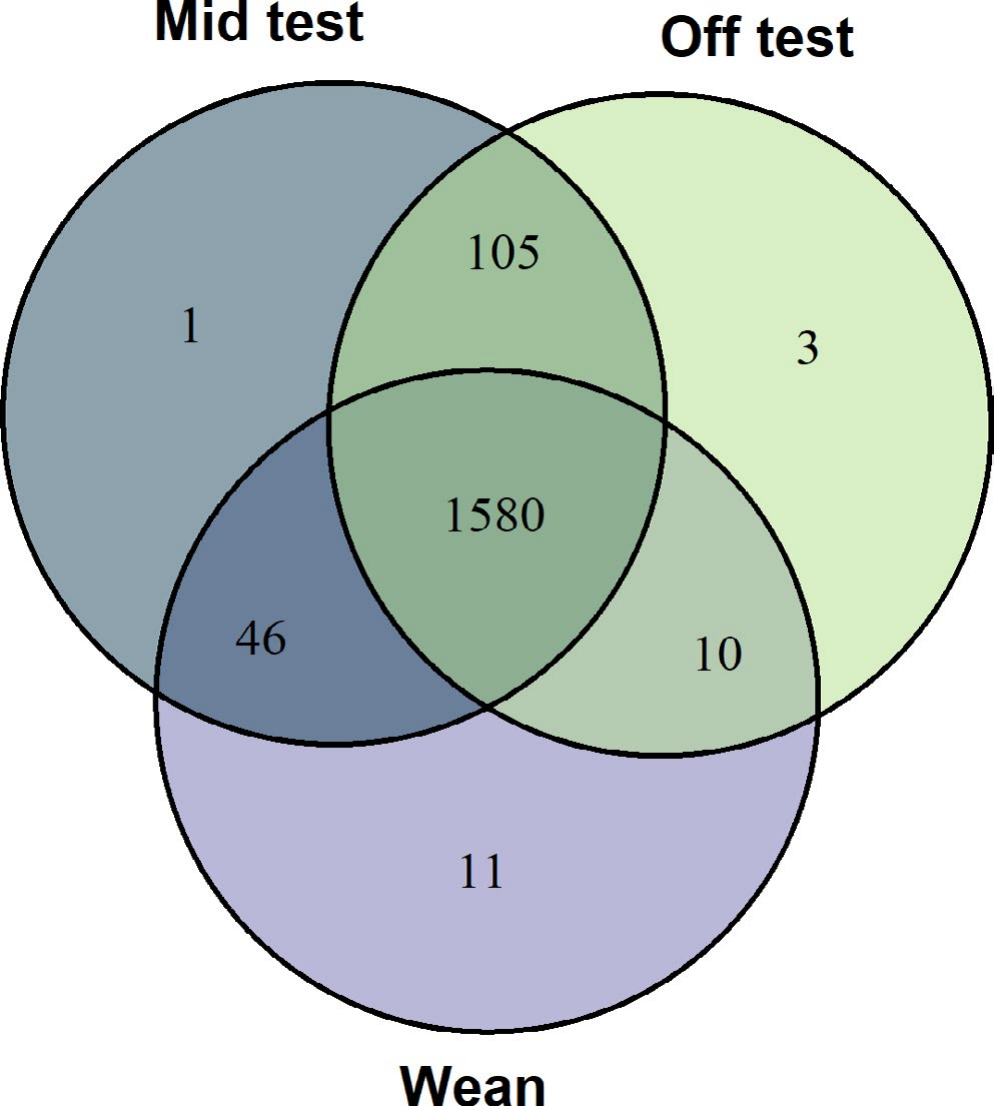
Venn diagram with the numbers of common operational taxonomic units (OTU) among different growth stages (Wean, Mid‐test and Off‐test) [Colour figure can be viewed at wileyonlinelibrary.com]

Mean and standard deviation for each meat quality and carcass composition trait are provided in Table 1. There were nine meat quality and six carcass composition traits. The number of individual samples with complete genotypic, phenotypic and microbiome information at each stage was 1,123, which was used for further analyses. The distribution of OTU at weaning, Mid‐test and Off‐test is given in Figure 1. Of a total 1,755 OTU, there were 1,580 OTU in common between weaning, Mid‐test and Off‐test. There were 1,685 OTU in common between Mid‐test and Off‐test, while between weaning and Mid‐test were 1,626 and between weaning and Off‐test were 1,590.

### 3.2 Microbiability estimates

**FIGURE 2:**
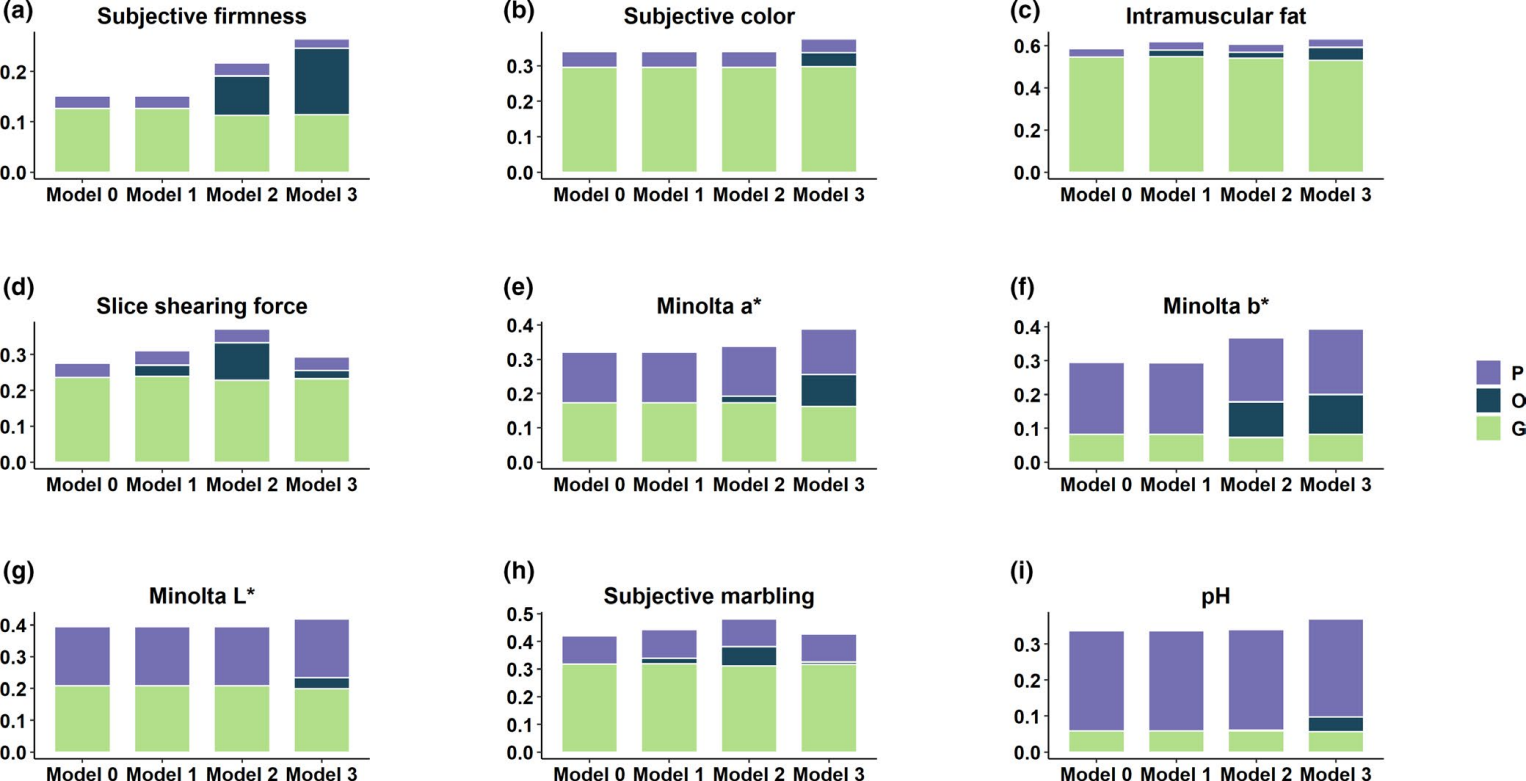
Proportion of variance explained by the inclusion of microbial (O), genomic (G) and pen (P) effect for meat quality traits. Model 0 contains genomic and pen effect as random effect, and Model 1, Model 2 and Model 3 contain microbial effect at weaning, Mid‐test and Off‐test in addition to genomic and pen effect [Colour figure can be viewed at wileyonlinelibrary.com]

**FIGURE 3:**
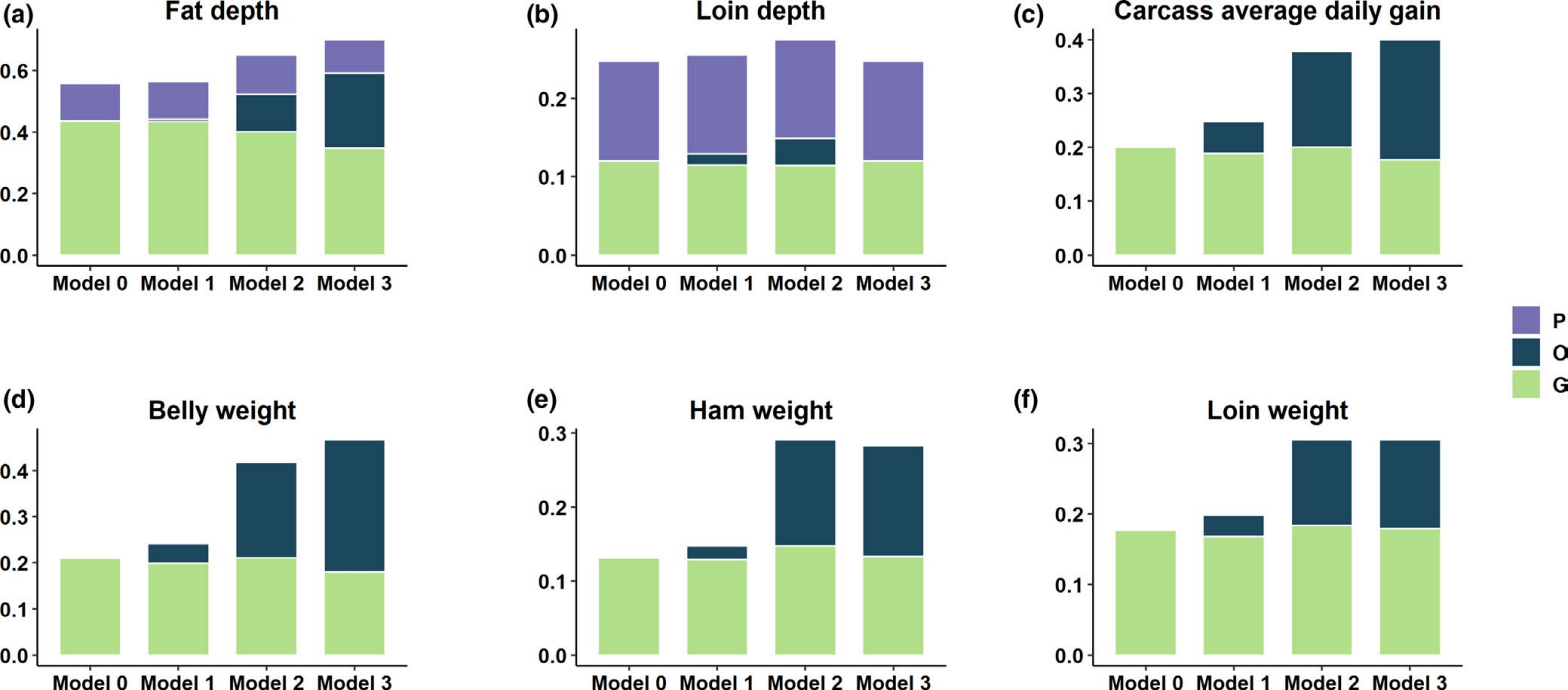
Proportion of variance explained by the inclusion of microbial (O), genomic (G) and pen (P) effect for carcass composition traits. Model 0 contains genomic and pen effect as random effect, and Model 1, Model 2 and Model 3 contain microbial effect at weaning, Mid‐test and Off‐test in addition to genomic and pen effect [Colour figure can be viewed at wileyonlinelibrary.com]

The proportion of variance explained by each random term for meat quality and carcass composition traits is presented in Figures 2 and 3, respectively. The estimates of heritability, microbiability and variance components along with their respective standard errors are provided in Table 2. The variance component estimates from the model which contain only the microbiome information and pen are also provided (Table S6). The results identified several traits with significant microbiability. Likelihood ratio tests showed that inclusion of genomic or microbiome information in the model performs significantly better (*p* < .001) than the null model (i.e., including all fixed effects and random effect of pen). The model containing both genomic and microbiome information was significantly better *(p < *.001) than the models containing genomic or microbiome information in addition to null model. Correlations between models' solutions were performed in order to have a proxy measure of the covariance between the host genomic and gut microbial effects; results are included in Tables S7 and S8.

**TABLE 2:** Variance components explained by the inclusion of microbial effect ($\sigma_{m}^{2}$), genomic effect ($\sigma_{a}^{2}$), pen effect ($\sigma_{\text{pen}}^{2}$) , residual effect ($\sigma_{e}^{2}$), microbiability (*m* ^2^) and heritability (*h* ^2^) in different models^a^

Traits	Parameter	Model 0	Model 1	Model 2	Model 3
IMF	$\sigma_{\text{pen}}^{2}$	0.04 ± 0.02	0.04 ± 0.02	0.04 ± 0.02	0.04 ± 0.02
	$\sigma_{m}^{2}$	–	0.03 ± 0.02	0.03 ± 0.02	0.06 ± 0.03
	$\sigma_{a}^{2}$	0.55 ± 0.08	0.55 ± 0.08	0.54 ± 0.08	0.53 ± 0.08
	$\sigma_{e}^{2}$	0.41 ± 0.05	0.38 ± 0.04	0.39 ± 0.05	0.37 ± 0.37
	*m* ^2^	–	0.03 ± 0.02	0.03 + 0.02	0.06 ± 0.02
	*h* ^2^	0.55 ± 0.05	0.54 ± 0.05	0.54 ± 0.05	0.53 ± 0.05
SMARB	$\sigma_{\text{pen}}^{2}$	0.08 ± 0.02	0.08 ± 0.02	0.08 ± 0.02	0.08 ± 0.02
	$\sigma_{m}^{2}$	–	0.02 ± 0.03	0.06 ± 0.02	0.007 ± 0.02
	$\sigma_{a}^{2}$	0.26 ± 0.08	0.26 ± 0.06	0.26 ± 0.06	0.26 ± 0.06
	$\sigma_{e}^{2}$	0.48 ± 0.04	0.46 ± 0.04	0.43 ± 0.05	0.47 ± 0.05
	*m* ^2^	–	0.02 ± 0.02	0.07 + 0.02	0.01 ± 0.02
	*h* ^2^	0.32 ± 0.05	0.32 ± 0.05	0.31 ± 0.05	0.32 ± 0.05
MINA	$\sigma_{\text{pen}}^{2}$	0.19 ± 0.04	0.18 ± 0.03	0.18 ± 0.03	0.17 ± 0.03
	$\sigma_{m}^{2}$	–	0.23E−04 ± 0.00	0.02 ± 0.04	0.12 ± 0.05
	$\sigma_{a}^{2}$	0.22 ± 0.06	0.22 ± 0.06	0.22 ± 0.06	0.22 ± 0.06
	$\sigma_{e}^{2}$	0.85 ± 0.06	0.85 ± 0.06	0.83 ± 0.05	0.77 ± 0.07
	*m* ^2^	–	0.00 ± 0.00	0.02 + 0.02	0.09 ± 0.02
	*h* ^2^	0.17 ± 0.05	0.17 ± 0.05	0.17 ± 0.05	0.16 ± 0.05
MINB	$\sigma_{\text{pen}}^{2}$	0.15 ± 0.03	0.15 ± 0.03	0.14 ± 0.03	0.13 ± 0.03
	$\sigma_{m}^{2}$	–	0.64E−05 ± 0.00	0.005 ± 0.01	0.08 ± 0.03
	$\sigma_{a}^{2}$	0.056 ± 0.03	0.056 ± 0.03	0.058 ± 0.03	0.056 ± 0.03
	$\sigma_{e}^{2}$	0.49 ± 0.03	0.49 ± 0.04	0.48 ± 0.04	0.42 ± 0.04
	*m* ^2^	–	0.00 ± 0.00	0.007 + 0.02	0.11 ± 0.04
	*h* ^2^	0.08 ± 0.04	0.08 ± 0.04	0.08 ± 0.04	0.08 ± 0.04
MINL	$\sigma_{\text{pen}}^{2}$	6.15 ± 1.16	6.15 ± 1.16	6.15 ± 1.16	6.15 ± 1.16
	$\sigma_{m}^{2}$	–	0.61E−05 ± 0.00	0.99E−05 ± 0.00	1.16 ± 1.15
	$\sigma_{a}^{2}$	6.90 ± 1.82	6.91 ± 1.82	6.91 ± 1.82	6.57 ± 1.78
	$\sigma_{e}^{2}$	20.05 ± 1.63	20.04 ± 1.52	20.04 ± 1.52	19.23 ± 1.81
	*m* ^2^	–	0.00 ± 0.00	0.00 + 0.00	0.03 ± 0.03
	*h* ^2^	0.21 ± 0.04	0.21 ± 0.05	0.21 ± 0.05	0.19 ± 0.05
PH	$\sigma_{\text{pen}}^{2}$	0.013 ± 0.002	0.013 ± 0.002	0.013 ± 0.002	0.013 ± 0.002
	$\sigma_{m}^{2}$	–	0.26E−05 ± 0.00	0.001 ± 0.007	0.002 ± 0.002
	$\sigma_{a}^{2}$	0.003 ± 0.001	0.003 ± 0.001	0.003 ± 0.001	0.003 ± 0.001
	$\sigma_{e}^{2}$	0.031 ± 0.002	0.031 ± 0.002	0.031 ± 0.002	0.031 ± 0.002
	*m* ^2^	–	0.00 ± 0.00	0.002 + 0.01	0.04 ± 0.03
	*h* ^2^	0.06 ± 0.04	0.06 ± 0.04	0.06 ± 0.04	0.06 ± 0.04
SCOL	$\sigma_{\text{pen}}^{2}$	0.014 ± 0.006	0.013 ± 0.006	0.014 ± 0.006	0.013 ± 0.006
	$\sigma_{m}^{2}$	–	0.41E−05 ± 0.00	0.40E−05 ± 0.00	0.012 ± 0.011
	$\sigma_{a}^{2}$	0.096 ± 0.02	0.096 ± 0.02	0.096 ± 0.02	0.097 ± 0.02
	$\sigma_{e}^{2}$	0.216 ± 0.018	0.215 ± 0.018	0.215 ± 0.018	0.204 ± 0.019
	*m* ^2^	–	0.00 ± 0.00	0.002 + 0.01	0.04 ± 0.03
	*h* ^2^	0.30 ± 0.06	0.30 ± 0.05	0.30 ± 0.06	0.30 ± 0.06
SFIRM	$\sigma_{\text{pen}}^{2}$	0.026 ± 0.029	0.026 ± 0.029	0.028 ± 0.029	0.022 ± 0.028
	$\sigma_{m}^{2}$	–	0.41E−05 ± 0.00	0.40E−05 ± 0.00	0.012 ± 0.011
	$\sigma_{a}^{2}$	0.134 ± 0.050	0.134 ± 0.050	0.120 ± 0.044	0.122 ± 0.046
	$\sigma_{e}^{2}$	0.904 ± 0.059	0.904 ± 0.059	0.834 ± 0.063	0.791 ± 0.068
	*m* ^2^	–	0.00 ± 0.00	0.08 + 0.03	0.13 ± 0.04
	*h* ^2^	0.13 ± 0.04	0.13 ± 0.05	0.11 ± 0.04	0.11 ± 0.04
SSF	$\sigma_{\text{pen}}^{2}$	0.52 ± 0.36	0.52 ± 0.36	0.50 ± 0.36	0.49 ± 0.36
	$\sigma_{m}^{2}$	–	0.41 ± 0.32	1.38 ± 0.59	0.30 ± 0.36
	$\sigma_{a}^{2}$	3.07 ± 0.74	3.12 ± 0.75	3.00 ± 0.73	3.03 ± 0.73
	$\sigma_{e}^{2}$	9.42 ± 0.70	8.99 ± 0.77	8.27 ± 0.78	9.20 ± 0.66
	*m* ^2^	–	0.03 ± 0.02	0.10 + 0.04	0.02 ± 0.02
	*h* ^2^	0.24 ± 0.05	0.24 ± 0.05	0.22 ± 0.05	0.23 ± 0.05
LD	$\sigma_{\text{pen}}^{2}$	6.55 ± 1.67	6.49 ± 1.66	6.42 ± 1.67	6.55 ± 1.67
	$\sigma_{m}^{2}$	–	0.71 ± 1.05	1.76 ± 1.58	0.29E−04 ± 0.00
	$\sigma_{a}^{2}$	6.15 ± 2.94	5.88 ± 2.36	5.88 ± 2.36	6.15 ± 2.93
	$\sigma_{e}^{2}$	38.53 ± 2.06	38.09 ± 2.05	37.20 ± 2.15	38.53 ± 2.06
	*m* ^2^	–	0.01 ± 0.02	0.03 + 0.03	0.00 ± 0.00
	*h* ^2^	0.12 ± 0.04	0.11 ± 0.05	0.12 ± 0.04	0.11 ± 0.04
FD	$\sigma_{\text{pen}}^{2}$	2.78 ± 0.62	2.77 ± 0.65	2.90 ± 0.67	2.37 ± 0.60
	$\sigma_{m}^{2}$	–	0.18 ± 0.48	2.71 ± 0.87	5.37 ± 1.05
	$\sigma_{a}^{2}$	9.84 ± 1.55	9.80 ± 1.56	9.00 ± 1.48	7.61 ± 1.34
	$\sigma_{e}^{2}$	9.99 ± 1.05	9.84 ± 1.14	7.83 ± 1.13	6.56 ± 1.05
	*m* ^2^	–	0.01 ± 0.02	0.12 + 0.04	0.25 ± 0.04
	*h* ^2^	0.44 ± 0.05	0.43 ± 0.05	0.40 ± 0.05	0.34 ± 0.05
CADG	$\sigma_{\text{pen}}^{2}$	0.78E−05 ± 0.00	0.72E−0 ± 0.00	0.61E−05 ± 0.00	0.59E−05 ± 0.00
	$\sigma_{m}^{2}$	–	0.31 ± 0.18	0.95 ± 0.26	1.18 ± 0.29
	$\sigma_{a}^{2}$	1.05 ± 0.23	0.98 ± 0.08	1.07 ± 0.29	0.94 ± 0.27
	$\sigma_{e}^{2}$	4.71 ± 0.29	4.63 ± 0.30	3.31 ± 0.26	3.18 ± 0.30
	*m* ^2^	–	0.06 ± 0.03	0.18 + 0.04	0.22 ± 0.05
	*h* ^2^	0.20 ± 0.05	0.18 ± 0.05	0.20 ± 0.05	0.18 ± 0.04
HAM	$\sigma_{\text{pen}}^{2}$	0.87E−07 ± 00	0.85E ± 0.00	0.72E−05 ± 0.00	0.73E−05 ± 0.0
	$\sigma_{m}^{2}$	–	0.10 ± 0.16	0.80 ± 0.25	0.83 ± 0.29
	$\sigma_{a}^{2}$	0.72 ± 0.27	0.70 ± 0.27	0.82 ± 0.28	0.73 ± 0.27
	$\sigma_{e}^{2}$	4.71 ± 0.29	4.65 ± 0.32	3.96 ± 0.33	3.96 ± 0.33
	*m* ^2^	–	0.02 ± 0.02	0.14 + 0.04	0.15 ± 0.05
	*h* ^2^	0.13 ± 0.05	0.13 ± 0.04	0.13 ± 0.05	0.13 ± 0.04
LOIN	$\sigma_{\text{pen}}^{2}$	0.82E−05 ± 0.0	0.80E−05 ± 0.00	0.70E−05 ± 00	0.70E−05 ± 00
	$\sigma_{m}^{2}$	–	0.10 ± 0.16	0.43 ± 0.16	0.44 ± 0.18
	$\sigma_{a}^{2}$	0.62 ± 0.18	0.58 ± 0.17	0.65 ± 0.18	0.64 ± 0.18
	$\sigma_{e}^{2}$	2.87 ± 0.18	2.79 ± 0.19	2.46 ± 0.21	2.45 ± 0.21
	*m* ^2^	–	0.03 ± 0.02	0.12 + 0.04	0.13 ± 0.05
	*h* ^2^	0.18 ± 0.05	0.17 ± 0.05	0.18 ± 0.05	0.18 ± 0.04
BEL	$\sigma_{\text{pen}}^{2}$	0.79E−05 ± 0.0	0.76E−05 ± 0.00	0.59E−05 ± 0.00	0.54E−05 ± 0.00
	$\sigma_{m}^{2}$	–	0.28 ± 0.20	1.37 ± 0.38	1.89 ± 0.38
	$\sigma_{a}^{2}$	1.34 ± 0.36	1.28 ± 0.35	1.39 ± 0.36	1.18 ± 0.33
	$\sigma_{e}^{2}$	5.09 ± 0.34	4.88 ± 0.36	3.45 ± 0.38	3.50 ± 0.37
	*m* ^2^	–	0.04 ± 0.03	0.20 + 0.04	0.29 ± 0.05
	*h* ^2^	0.21 ± 0.05	0.19 ± 0.05	0.21 ± 0.05	0.18 ± 0.04

Abbreviations: BEL, belly weight; CADG, carcass average daily gain; FD, fat depth; HAM, ham weight; IMF, intramuscular fat percent; LD, loin depth; LOIN, loin weight; MINA, Minolta a*; MINB, Minolta b*; MINL, Minolta L*; PH, ultimate pH; SCOL, subjective colour score; SFIRM, subjective firmness score; SMARB, subjective marbling score; SSF, slice shear force.

^a^ Model 0 contains genomic and pen effect as random effect, and Model 1, Model 2 and Model 3 contain microbial effect at weaning, Mid‐test and Off‐test, respectively, in addition to genomic and pen effect.

The microbiability of carcass composition traits were higher than those of meat quality traits. In all cases, microbiability estimates for both meat quality and carcass composition traits at weaning were negligible and ranged from zero for several traits to a maximum of 0.06 ± 0.03 (estimate ± *SE*) for CADG. Three of the nine meat quality traits investigated showed significant microbiability at Mid‐test, with estimates of 0.07 ± 0.02 for SMARB, 0.08 ± 0.03 for SFIRM and 0.10 ± 0.04 for SSF. At Off‐test, four meat quality traits had significant microbiability, with estimates of 0.06 ± 0.02 for IMF, 0.09 ± 0.02 for MINA, 0.11 ± 0.04 for MINB and 0.13 ± 0.04 for SFIRM. For carcass composition traits, we found that five out of six traits were significantly affected by microbiome at Mid‐test and Off‐test. The microbiability of carcass composition traits at Mid‐test ranged from 0.12 ± 0.04 for LOIN and FD to 0.20 ± 0.04 for BEL. The microbiability of carcass composition traits at Off‐test ranged from 0.13 ± 0.05 for LOIN to 0.29 ± 0.05 for BEL. In our study, the microbiability was not significant for LD. In most of the cases, we did not find significant microbiability estimates at weaning; however, microbiability estimates at Mid‐test and Off‐test were significant. This might have several causes including the sudden change of microbiome composition shortly after the diet switch occurring at weaning as well as other environmental factors like, stress. Greater microbiability estimates indicate that the microbial composition is more informative for the phenotype. Based on our microbiability estimates, microbial composition at Off‐test are important predictors of carcass composition traits compared to Mid‐test and Wean because the microbiability estimates for majority of the traits were higher at Off‐test than the Mid‐test and Wean. To our knowledge, this is the first attempt to obtain microbiability estimates for meat quality and carcass composition traits. We did not find any literature on swine to compare the estimates with previous research. However, Buitenhuis et al. (2019), Wen et al. (2019) and Rothschild et al. (2018) reported significant microbiability for fatty acids in milk of Holstein cattle, abdominal fat of chicken and body mass index and glycaemic index in human, respectively. Based on these literatures and our finding of high microbiability estimate of fat‐related traits, it was evident that gut microbiota has impact on lipid metabolism and fat deposition in swine. Our results suggest that later measures of microbial composition might be more informative for selection purposes, but further research would be needed to clarify this aspect.

Among meat quality traits, microbial variance explained a larger proportion of phenotypic variance than additive genetic for SFIRM and MINB at Off‐test (Figure 2). Among carcass composition traits, BEL, HAM and CADG at Off‐test had higher proportion of phenotypic variation explained by microbiome than by additive genetic (Figure 3). These results indicated that a significant proportion of total variance is explained by the microbiome, in some cases larger than the additive genetics and that prediction for these traits could be improved by accounting for the effect of variability in gut microbiome composition. The variation in gut microbiome could be fitted as the systematic environmental effect in model. Overall, to utilize the microbiome variability in the host–microbiome system, causal relationships among all systems should be established and fitted in the model. For example, the environmental effect on microbial composition, effect of host genes on microbial composition, direct effect of microbial composition on phenotypes and host genomic effects on phenotypes as mediated by microbiome should be fitted in the model.

In the current study, we observed a decrease in genomic heritability for most of the carcass composition traits at Off‐test when microbiome information was added. The decrease in heritability ranged from 1% for LD to approximately 10% for FD. At Mid‐test, the decrease in heritability ranged from 0% for CADG, BEL, HAM and LOIN to 4% for FD. No change in genomic heritability was observed at weaning. The decrease in heritability for FD was similar to that found by Lu et al. (2018) for similar traits. He et al. (2016) also reported the significant contribution of microbiome for porcine fatness. The results suggested that part of the resemblance among individuals captured by genetic effects in breeding values prediction, might be in fact a resemblance among microbial composition and genetic parameters might not be accurate.

In contrast, for most of the meat quality traits considered, the inclusion of microbial composition did not affect the estimates of genomic heritability, thus suggesting that at least for meat quality traits, gut microbial composition is mostly an environmental factor. The decrease in genomic heritability when we included the microbiome composition in the models was previously observed by Sandoval‐Motta et al. (2017) who reported the possibility of overestimation of heritability values with the use of genetic similarities by kinship information. The authors also suggested that inclusion of genetic diversity of individual microbiome will most likely increase the accuracy of heritability of various traits. The heritability and microbiability estimation of daily gain, feed intake and feed conversion ratio in swine (Camarinha‐Silva et al., 2017) and methane emission in cattle (Difford et al., 2018) strongly suggested a significant contribution of microbiome to the total variation in the complex phenotypes of livestock. In human, Richards et al. (2019) reported that host gene effects are affected by the microbiome composition. These previous studies agreed with our results. Our results also agreed with the concept of “hologenome” of evolution (Zilber‐Rosenberg & Rosenberg, 2008), where the animal or plant along with associated microorganisms are the unit of selection in evolution. Based on our results, pigs act as holobiont for FD at Off‐test as there is change in heritability with the inclusion of microbiome information and change in microbiability with the inclusion of genomic information.

### 3.3 Microbial correlation among traits

In the discussion of correlation, we only focus on microbial correlations. Genomic correlations are only discussed if the genomic correlations changed due to inclusion of microbiome information in the model. The genomic correlations among traits without inclusion of microbiome in the model are presented in Tables S9–S12.

#### 3.3.1 Correlations among meat quality and carcass composition traits at Mid‐test

Overall, there were three meat quality traits and five carcass composition traits having variance of microbiome composition greater than 3%. Microbial correlations among meat quality and carcass composition traits at Mid‐test are presented in Table 3. Most of the microbial correlations were significant. SMARB was moderately positively correlated (0.46 ± 0.24) with FD. This suggested that shifting of microbiota for high marbled meat would result in higher FD. Shear force is the measure of tenderness. In this study, the microbial composition of SSF was highly negatively correlated with SMARB, SFIRM, FD, CADG, LOIN and BEL which ranged from −0.93 ± 0.11 for SSF and SFIRM to −0.50 ± 0.25 for SSF and LOIN. High positive correlations of SFIRM were found with CADG, HAM, LOIN and BEL which ranged from 0.58 ± 0.26 between SFIRM and LOIN to 0.87 ± 0.16 between SFIRM and BEL. There were moderate to high correlations of microbial composition of FD with CADG, HAM, LOIN and BEL which ranged from 0.44 ± 0.21 between FD and LOIN to 0.74 ± 0.11 between FD and BEL. High positive correlations were found between CADG and HAM, LOIN and BEL. BEL was highly positively correlated with HAM (0.96 ± 0.03) and LOIN (0.94 ± 0.06). We did not find any other estimates to compare our values with microbial correlation between meat quality and carcass composition traits.

**TABLE 3:** Estimates of microbial correlation (above diagonal) and genomic correlation (below diagonal) at Mid‐test among meat quality and carcass composition traits

	SMARB	SFIRM	SSF	FD	CADG	HAM	LOIN	BEL
SMARB		0.39 ± 0.33	**−0.72 ± 0.28**	**0.46 ± 0.24**	−0.21 ± 0.28	−0.27 ± 0.29	−0.34 ± 0.32	−0.02 ± 0.26
SFIRM	**0.42 ± 0.18**		**−0.93 ± 0.11**	NC	**0.86 ± 0.17**	**0.62 ± 0.24**	**0.58 ± 0.26**	**0.87 ± 0.16**
SSF	0.08 ± 0.16	−0.23 ± 0.21		**−0.70 ± 0.21**	**−0.68 ± 0.22**	−0.45 ± 0.25	**−0.50 ± 0.25**	**−0.55 ± 0.24**
FD	**0.22 ± 0.11**	NC	**−0.44 ± 0.13**		**0.68 ± 0.15**	**0.50 ± 0.19**	**0.44 ± 0.21**	**0.74 ± 0.11**
CADG	0.02 ± 0.17	0.03 ± 0.23	0.19 ± 0.18	0.21 ± 0.15		**0.98 ± 0.02**	**0.95 ± 0.03**	**0.98 ± 0.01**
HAM	−0.13 ± 0.18	0.11 ± 0.24	0.27 ± 0.20	0.01 ± 0.15	**0.67 ± 0.11**		NE	**0.96 ± 0.03**
LOIN	−0.09 ± 0.17	0.10 ± 0.23	0.11 ± 0.18	−0.14 ± 0.15	**0.69 ± 0.09**	**0.53 ± 0.11**		**0.94 ± 0.06**
BEL	0.31 ± 0.17	0.35 ± 0.23	0.18 ± 0.15	**0.57 ± 0.11**	**0.79 ± 0.06**	**0.42 ± 0.17**	**0.42 ± 0.15**	

Note

Numbers in bold are significant.

Abbreviations: BEL, belly weight; CADG, carcass average daily gain; FD, fat depth; HAM, ham weight; LOIN, loin weight; NC, not converged; NE, not estimable; SFIRM, subjective firmness score; SMARB, subjective marbling score; SSF, slice shear force.

#### 3.3.2 Correlation between meat quality traits and carcass composition traits at off‐test

There were six meat quality traits and five carcass composition for which variance of microbiome composition was greater than 3%. The microbial and genomic correlations among meat quality traits at Off‐test are presented in Table 4. pH had high positive microbial correlation (0.90 ± 0.25) with SCOL and SFIRM (0.73 ± 0.35). This is in partial agreement with results from Ratzke and Gore (2018), that reported the specific bacteria which is responsible for building lactic acid in the muscle results in the anaerobic breakdown of glucose and glycogen, which eventually loosens the myofibril, thus scattering more light making the muscle pale (Walters, 1975). Furthermore, increasing pH causes swelling of myofibrils (Huff‐Lonergan & Lonergan, 2005) which ultimately makes the muscle firmer. High positive microbial correlation was found between IMF and SFIRM (0.91 ± 0.17), MINA (0.55 ± 0.28) and MINB (0.75 ± 0.27). This agrees with Fang, Xiong, Su, Huang, and Chen (2017) who reported that gut bacteria involved in energy metabolism and IMF in pig also regulate the muscle composition and muscle fibres. Higher microbial correlation of IMF with minolta colour measurements and SFIRM indicated that microbial composition increasing IMF would make the muscle paler and firmer. High microbial correlation of MINA and MINB (0.78 ± 0.16) suggests that microbiota associated for redness of meat are also associated with the yellowness in the meat. This agreed with Kim et al. (2010) who reported the positive correlation of yellowness and redness in the muscle of pig.

**TABLE 4:** Estimates of microbial correlation (above diagonal) and genomic correlation (below diagonal) at Off‐test among meat quality traits

	SCOL	IMF	SFIRM	MINA	MINB	PH
SCOL		−0.28 ± 0.57	0.07 ± 0.31	0.29 ± 0.44	−0.26 ± 0.39	**0.90 ± 0.25**
IMF	−0.22 ± 0.13		**0.91 ± 0.17**	**0.55 ± 0.28**	**0.75 ± 0.27**	0.10 ± 0.47
SFIRM	0.18 ± 0.19	0.29 ± 0.17		0.26 ± 0.27	0.12 ± 0.26	**0.73 ± 0.35**
MINA	**0.45 ± 0.16**	**0.29 ± 0.14**	−0.53 ± 0.28		**0.78 ± 0.16**	0.33 ± 0.36
MINB	**−0.94 ± 0.22**	**0.78 ± 0.16**	−0.03 ± 0.32	−0.10 ± 0.27		0.38 ± 0.38
PH	0.13 ± 0.50	−0.18 ± 0.25	0.44 ± 0.36	−0.04 ± 0.33	−0.47 ± 0.42	

Note

Numbers in bold are significant.

Abbreviations: IMF, intramuscular fat per cent; MINA, Minolta a*; MINB, Minolta b*; PH, ultimate pH; SCOL, subjective colour score; SFIRM, subjective firmness score.

The microbial and genomic correlations among carcass composition traits at Off‐test are presented in Table 5. The microbial correlation of carcass composition traits was highly and positively correlated to each other ranging from 0.55 ± 0.17 between FD and LOIN to 0.97 ± 0.02 between CADG and HAM. McCormack et al. (2018) reported a positive correlation between gut microbiota and feed efficiency in swine. Gut microbiota has significant association with feed intake, final body weight (Kubasova et al., 2018) and growth traits (Ramayo‐Caldas et al., 2016). All these studies suggested that microbial composition has considerable effects on many carcass composition traits, with positive correlations between them. These high correlations indicated that all the traits could be simultaneously improved through the same microbial composition.

**TABLE 5:** Estimates of microbial correlation (above diagonal) and genomic correlation (below diagonal) at Off‐test among carcass composition traits

	FD	CADG	HAM	LOIN	BEL
FD		**0.71 ± 0.11**	**0.59 ± 0.16**	**0.55 ± 0.17**	**0.94 ± 0.05**
CADG	0.14 ± 0.15		**0.97 ± 0.02**	**0.91 ± 0.05**	**0.94 ± 0.03**
HAM	−0.10 ± 0.17	**0.63 ± 0.13**		NE	**0.87 ± 0.06**
LOIN	−0.13 ± 0.15	**0.67 ± 0.10**	**0.54 ± 0.19**		**0.82 ± 0.08**
BEL	**0.49 ± 0.13**	**0.78 ± 0.07**	0.34 ± 0.19	**0.40 ± 0.16**	

Note

Numbers in bold are significant.

Abbreviations: BEL, belly weight; CADG, carcass average daily gain; FD, fat depth; HAM, ham weight; LOIN, loin weight; NE, non‐estimable.

The microbial correlations for meat quality traits and carcass composition traits at Off‐test are presented in Table 6. Intramuscular fat was highly correlated with FD (0.90 ± 0.14) and BEL (0.73 ± 0.18). Firmness score was positively correlated with BEL (0.50 ± 0.18). Moderate positive correlation was found between MINA and BEL (0.41 ± 0.21), and high positive correlation was found between MINA and FD (0.53 ± 0.18), and MINA and CADG (0.66 ± 0.17). Minolta b* had moderate positive correlation with FD (0.43 ± 0.19) and high positive correlation with CADG (0.58 ± 0.18), suggesting that increase in microbiota for lean meat and high daily gain of carcass would make the meat more yellowish.

**TABLE 6:** Estimates of microbial correlation between meat quality traits and carcass composition traits at Off‐test

	FD	CADG	HAM	LOIN	BEL
SCOL	−0.29 ± 0.37	−0.09 ± 0.35	0.16 ± 0.38	−0.25 ± 0.35	−0.32 ± 0.37
IMF	**0.90 ± 0.14**	0.43 ± 0.33	0.29 ± 0.27	0.21 ± 0.30	**0.73 ± 0.18**
SFIRM	NE	0.31 ± 0.19	0.18 ± 0.24	−0.01 ± 0.20	**0.50 ± 0.18**
MINA	**0.53 ± 0.18**	**0.66 ± 0.17**	0.11 ± 0.27	0.08 ± 0.30	**0.41 ± 0.21**
MINB	**0.43 ± 0.19**	**0.58 ± 0.18**	0.12 ± 0.25	−0.13 ± 0.28	0.35 ± 0.20
PH	0.17 ± 0.31	0.27 ± 0.35	NC	NC	0.11 ± 0.32

Note

Numbers in bold are significant.

Abbreviations: BEL, belly weight; CADG, carcass average daily gain; FD, fat depth; HAM, ham weight; LOIN, loin weight; IMF, intramuscular fat per cent; MINA, Minolta a*; MINB, Minolta b*; PH, ultimate pH; SCOL, subjective colour score; SFIRM, subjective firmness score. NC, not converged; NE, non estimable.

### 3.4 Change in genomic correlation with the inclusion of microbiome information

In this study, we observed a decrease in genomic correlations among meat quality and carcass composition traits when microbiome information was included in the model. The genomic correlations without the inclusion of microbial effect in model are provided in Tables S9–S12. At Mid‐test, the decrease in genomic correlation ranged from 0% among majority of meat quality traits to 18% for BEL and LOIN. The genomic correlation of BEL with FD and HAM decreased by 5% and 16%, respectively. The genomic correlation of FD with SMARB and SSF decreased by 7% and 4%, respectively.

At Off‐test, the genomic correlation between PH and SCOL (0.91 ± 0.29), SFIRM and IMF (0.36 ± 0.15), FD and CADG (0.27 ± 0.13), and BEL and HAM (0.58 ± 0.19) became non‐significant with the inclusion of microbial effect. Among carcass traits, the decrease in genomic correlation ranged from 1% between BEL and CADG to 30% between BEL and LOIN. The genomic correlation of BEL with FD, CADG with HAM, CADG with LOIN, FD with IMF, FD with MINB, BEL with IMF and BEL with SFIRM decreased by 13%, 4%, 2%, 9%, 6%, 13% and 8%, respectively. Among meat quality and carcass traits, the decrease in genomic correlations ranged from 1% for FD and SFIRM to 9% for BEL and IMF. We observed a decrease in genomic correlations with the inclusion of microbial effect, particularly of any other traits with fat‐related traits (e.g., BEL, FD, IMF). This could be due to the greater influence of gut microbiome on fat deposition. The holobiont behaviour of FD at Off‐test and Mid‐test might also explain the decrease in genomic correlations with other traits. Furthermore, we observed that there was a decrease in genomic correlation for those traits which had higher microbial correlation. High microbial correlations among different traits suggested that genomic correlations among traits are partially contributed by the correlations among the gut microbiota composition. The covariance among microbiome for different traits might have contributed to the genetic covariance and hence the genomic correlation. We observed that the decrease in the genomic correlation was higher at Off‐test than at Mid‐test. This was due to high variability accounted by microbiome composition at Off‐test in comparison to Mid‐test.

This is the first study to evaluate the variance accounted by microbiome and estimate the microbial correlations for meat quality and carcass traits in swine. So, we have explored the model sequentially, first with inclusion of genomic information and then addition of microbiome effect at different stages to evaluate the change in variance components. In cattle, Difford et al. (2018) and Ramayo‐Caldas et al. (2020) fitted the genomic and microbiome information simultaneously and reported that both of them were jointly associated with methane emissions.

Variance component estimates of different random effects with inclusion of interaction of genotype‐by‐microbiome in the model are recommended for future studies.

## 4 CONCLUSIONS

This study was conducted on crossbred pigs to investigate the impact of intestinal microbiota through different stages (Wean, Mid‐test and Off‐test) of production. To our knowledge, this study is the first attempt to investigate the impact of microbiome on the meat quality and carcass composition traits at a large scale in swine. The contribution of microbiome to all traits was significant although it varied over time with an increase from weaning to Off‐test for most of the traits. Adding microbiome information did not affect the estimates of genomic heritability of meat quality traits but changed the estimate of carcass composition traits suggesting that portion of genomic variance was contributed by gut microbiome. A better understanding of microbial composition could aid the improvement of complex traits, particularly the carcass composition traits in swine by inclusion of microbiome information in the genetic evaluation process. High microbial correlations were found among different traits, particularly with traits related to fat deposition. Adding microbiome information decreased the genomic correlation for those traits which had higher microbial correlation suggesting that portion of genomic correlation was due to genetic covariance among microbiome composition affecting those traits. Based on the results, we can conclude that microbial composition could be altered to improve a given trait. Further research into causal mechanisms between microbial profiles, host genetic make‐up and phenotypic performance is needed to incorporate the microbiome information in the genetic evaluation process. The estimated parameters provide a reference value for further research on gut microbial contribution to complex phenotypes in pigs.

## CONFLICT OF INTEREST

The authors declare no conflict of interest.

## Supporting information

TableS1‐S12

## Data Availability

The data that support the findings of this study are available from MATATU, but restrictions apply to the availability of these data, which were used under licence for the current study, and so are not publicly available. Data are however available from the authors upon reasonable request and with permission of MATATU.
