# Supplementary material for: Microbiability of meat quality and carcass composition traits in swine
Source: J Anim Breed Genet. 2020 Sep 26;138(2):223–36. doi: 10.1111/jbg.12504 (PMC7891674; doi:10.1111/jbg.12504)
Supplement: Supplementary file 1 — TableS1‐S12 [file JBG-138-223-s001.pdf]

Table S1. Diet formulae and their nutritional values

	*Nursery 3	Nursery 4	GF-1	GF-2		GF-3		GF-4		GF-5		GF-6		GF-7
	Barrow/Gilt	Barrow/Gilt	Barrow/Gilt	Barrow	Gilt	Barrow	Gilt	Barrow	Gilt	Barrow	Gilt	Barrow	Gilt	Barrow/Gilt
Ingredient														
Corn	660.60	861.88	800.62	1020.46	1013.36	1236.71	1204.82	1382.01	1335.94	1481.33	1435.26	1530.99	1499.03	1534.50
Corn germ meal	48.01	341.84	564.17	464.72	467.93	366.89	381.32	301.16	322.00	256.23	277.07	233.76	248.22	232.18
Soybean meal	326.70	594.00	490.79	394.42	397.53	299.63	313.60	235.93	256.12	192.39	212.59	170.62	184.63	169.09
Fat - yellow grease (post-pellet)			64.46	47.08	47.64	29.99	32.51	18.50	22.14	10.65	14.29	6.72	9.25	6.45
Limestone		30.20	27.99	25.81	25.88	23.66	23.97	22.21	22.67	21.23	21.69	20.73	21.05	20.70
Pelleting aid			10.00	10.00	10.00	10.00	10.00	10.00	10.00	10.00	10.00	10.00	10.00	10.00
L-Lysine HCl (98%)		8.91	9.65	8.17	8.22	6.71	6.93	5.74	6.05	5.07	5.38	4.73	4.95	4.71
Salt		11.17	9.14	9.13	9.13	9.12	9.13	9.12	9.12	9.11	9.12	9.11	9.11	9.11
Fat - yellow grease	41.55	13.86	7.00	7.00	7.00	7.00	7.00	7.00	7.00	7.00	7.00	7.00	7.00	7.00
Monocalcium phosphate (21%)		18.13	5.51	4.52	4.55	3.54	3.69	2.89	3.09	2.44	2.65	2.21	2.36	2.20
HMTBa		2.26	4.50	3.29	3.33	2.09	2.27	1.29	1.55	0.74	1.00	0.47	0.65	0.45
L-Threonine (98%)		2.07	2.35	1.78	1.80	1.23	1.31	0.85	0.97	0.60	0.72	0.47	0.55	0.46
Trace mineral premix		1.98	2.00	1.87	1.87	1.73	1.75	1.64	1.67	1.58	1.61	1.55	1.57	1.55
Phytase 2500		1.90	0.80	0.76	0.76	0.73	0.73	0.70	0.71	0.68	0.69	0.67	0.68	0.67
Vitamin premix		0.99	0.60	0.57	0.57	0.55	0.55	0.53	0.53	0.52	0.52	0.51	0.51	0.51
Copper chloride (58%)		0.68	0.43	0.43	0.43	0.43	0.43	0.43	0.43	0.43	0.43	0.43	0.43	0.43
Nursery basemix	791.99													
DDGS	111.15	76.52												
Mecadox 2.5 (g/lb)	20	20												
Zinc oxide (72%)		6.93												
Organic acidifier		5.94												
Carbohydrase		0.74												
Total:	2000	2000	2000	2000	2000	2000	2000	2000	2000	2000	2000	2000	2000	2000

Nutrient	Units	*Nursery 3	Nursery 4	GF-1	GF-2 Barrow	GF-2 Gilt	GF-3 Barrow	GF-3 Gilt	GF-4 Barrow	GF-4 Gilt	GF-5 Barrow	GF-5 Gilt	GF-6 Barrow	GF-6 Gilt	GF-7
Metabolizable energy	Kcal/lb	1500	1519.371	1460.004	1460.18	1460.17	1460.35	1460.33	1460.47	1460.43	1460.55	1460.51	1460.59	1460.56	1460.59
Crude protein	%	20.103	22.561	21.281	18.59	18.68	15.95	16.34	14.17	14.74	12.96	13.52	12.35	12.74	12.31
Cystine, Dig	%	0.293	0.272	0.244	0.22	0.22	0.20	0.20	0.19	0.19	0.18	0.18	0.17	0.18	0.17
Isoleucine, Dig	%	0.675	0.786	0.715	0.62	0.62	0.52	0.53	0.45	0.47	0.41	0.43	0.39	0.40	0.38
Lysine, Total	%	1.405	1.521	1.454	1.23	1.24	1.01	1.05	0.87	0.91	0.77	0.81	0.72	0.75	0.71
Lysine, Dig	%	1.25	1.34	1.27	1.07	1.08	0.87	0.90	0.74	0.78	0.65	0.69	0.61	0.64	0.60
Leucine, Dig	%	1.469	1.586	1.439	1.31	1.31	1.18	1.20	1.09	1.12	1.03	1.06	1.00	1.02	1.00
Met + Cys, Dig	%	0.707	0.765	0.725	0.62	0.62	0.51	0.53	0.44	0.47	0.40	0.42	0.37	0.39	0.37
Threonine, Dig	%	0.76	0.804	0.762	0.65	0.65	0.54	0.55	0.46	0.48	0.41	0.43	0.38	0.40	0.38
Tryptophan, Dig	%	0.223	0.232	0.216	0.18	0.18	0.15	0.16	0.13	0.14	0.12	0.12	0.11	0.11	0.11
Valine, Dig	%	0.826	0.871	0.826	0.72	0.72	0.62	0.63	0.55	0.57	0.50	0.52	0.47	0.49	0.47
Phosphorus	%	0.728	0.689	0.557	0.50	0.50	0.44	0.45	0.41	0.42	0.38	0.39	0.37	0.38	0.37
P, Available	%	0.569	0.4	0.3	0.27	0.27	0.24	0.24	0.21	0.22	0.20	0.21	0.19	0.20	0.19
Calcium	%	0.809	0.896	0.7	0.63	0.63	0.57	0.58	0.52	0.54	0.49	0.51	0.48	0.49	0.48
Moisture	%	13.735	13.035	13.172	13.60	13.58	14.01	13.95	14.29	14.20	14.48	14.39	14.58	14.52	14.58
Crude fat	%	5.476	3.03	5.458	4.82	4.84	4.18	4.28	3.76	3.89	3.47	3.60	3.32	3.42	3.31
Crude fiber	%	2.117	3.18	3.486	3.17	3.18	2.85	2.90	2.64	2.71	2.50	2.57	2.43	2.47	2.42
ADF	%	2.92	5.01	5.367	4.86	4.88	4.37	4.44	4.03	4.14	3.81	3.91	3.69	3.76	3.68
NDF	%	6.507	12.75	15	13.58	13.63	12.19	12.40	11.26	11.55	10.62	10.91	10.30	10.50	10.28

* Nursery 3 and Nursery 4 are the nursery facilities where the pigs were kept after weaning. GF1-7 are the groups where grow-finish diets were provided. Animals were grouped into different place within the facility based on sex and live weight.

Table S2. Vaccinations

Condition	Timing
Mycoplasma Hyopneumoniae	Processing (~4 days old)
Porcine Circovirus Type 2 (PCV2)	Weaning
Porcine Respiratory Syndrome (PRRS)	10-14 days post-weaning
Porcine Circovirus<br>Mycoplasma Hyopneumoniae	10-17 days post PRRS vaccination
Ileitis<br>Erysipelas	~6 weeks post-weaning

Table S3. Injectable medications

Condition	Product	Timing
Respiratory, Diarrhea, Lameness	Excede	Weaning to 8 weeks post-weaning
Respiratory	Biomycin 200	8-14 weeks post-weaning
Respiratory	Lincocin 300	14 weeks post-weaning to end of study
Diarrhea, Lameness	Lincocin 300	8 weeks post weaning to end of study
Lameness	Dexamethasone	Weaning to 14 weeks post-weaning

Table S4. Water medications

Condition	Product	Timing
Diarrhea	Neomycin	Weaning
Respiratory, Diarrhea	Oxytetracycline (OTC), Denagard	As needed
Respiratory, Diarrhea, Lameness	Linco Soluble	As needed

Table S5. Distribution of samples across families, sex, and time points

Family	Female			Male			Total
	Weaning	Mid-test	Off-test	Weaning	Mid-test	Off-test	
1	22	23	22	20	20	20	127
2	19	25	24	23	23	21	135
3	20	23	22	20	23	23	131
4	23	24	23	21	23	23	137
5	21	18	21	15	15	15	105
6	21	24	24	23	25	25	142
7	18	22	21	19	20	19	119
8	20	25	25	23	24	23	140
9	21	24	25	25	25	26	146
10	22	25	25	23	24	22	141
11	21	22	23	24	24	23	137
12	20	21	20	20	22	22	125
13	23	25	24	21	23	21	137
14	24	26	25	22	21	21	139
15	23	23	23	25	25	24	143
16	19	24	23	24	25	25	140
17	19	20	21	22	23	23	128
18	23	23	22	23	23	23	137
19	22	26	26	20	19	19	132
20	22	25	22	24	26	20	139
21	18	21	21	18	19	19	116
22	21	25	23	23	22	24	138
23	19	23	21	19	22	20	124
24	22	25	25	23	24	23	142
25	24	27	27	20	23	23	144
26	22	24	25	23	23	24	141
27	23	26	27	21	24	24	145
28	24	26	25	25	20	23	143

Table S6: Variance components explained by the inclusion of microbial effect (σ_m^2), pen effect (σ_{pen}^2), residual (σ_e^2) and microbiability (m^2) at different stages of production when only microbiome information was included in the model

Traits[2]	Parameter	Weaning	Mid test	Off test
LD	σ_{pen}^2	7.35±1.71	7.30±1.71	7.47±1.71
	σ_m^2	1.16±1.19	2.27±1.69	2.3E-05±0.00
	σ_e^2	42.52±2.35	41.62±2.48	43.56±2.17
	m^2	0.03±0.02	0.04±0.03	0.00±0.00
FD	σ_{pen}^2	4.33±0.79	4.49±0.78	3.76±0.71
	σ_m^2	0.38±0.57	3.64±1.03	6.96±1.24
	σ_e^2	17.59±1.01	14.29±1.01	11.54±0.97
	m^2	0.02±0.02	0.16±0.04	0.31±0.05
CADG	σ_{pen}^2	4.86E-06±0.00	4.86E-07±0.00	4.86E-07±0.00
	σ_m^2	0.36±0.19	0.97±0.27	1.20±0.3
	σ_e^2	4.80±0.25	4.27±0.26	4.03±0.27
	m^2	0.07±0.03	0.19±0.04	0.23±0.09
HAM	σ_{pen}^2	2.09E-08±0.00	2.63E-07±0.00	2.57E-07±0.00
	σ_m^2	0.08±0.01	0.41±0.27	0.50±0.02
	σ_e^2	2.89±0.02	2.60±0.26	2.52±0.02
	m^2	0.03±0.03	0.13±0.04	0.16±0.05
LOIN	σ_{pen}^2	4.24E-06±0.00	3.07E-07±0.00	3.03E-07±0.00
	σ_m^2	0.19±0.12	0.41±0.16	0.43±0.02
	σ_e^2	3.27±0.17	3.07±0.18	3.06±0.18
	m^2	0.06±0.03	0.12±0.04	0.14±0.05
BEL	σ_{pen}^2	4.71E-06±0.00	3.95E-07±0.00	3.64E-07±0.00
	σ_m^2	0.29±0.12	1.18±0.35	1.47±0.39
	σ_e^2	4.69±0.17	3.95±0.31	3.56±0.32
	m^2	0.06±0.03	0.22±0.04	0.29±0.05
IMF	σ_{pen}^2	0.12±0.03	0.10±0.03	0.11±0.03
	σ_m^2	0.04±0.03	0.05±0.03	0.10±0.04
	σ_e^2	0.80±0.05	0.79±0.05	0.74±0.05
	m^2	0.04±0.03	0.05±0.03	0.11±0.04
SMARB	σ_{pen}^2	0.11±0.03	0.10±0.03	0.11±0.03
	σ_m^2	6E-08±0.00	0.05±0.03	0.02±0.02

	σ_e^2	0.69±0.03	0.64±0.04	0.67±0.04
	m^2	0.00±0.00	0.07±0.03	0.03±0.02
MINA	σ_{pen}^2	0.25±0.05	0.24±0.04	0.22±0.03
	σ_m^2	9E-08±0.00	0.03±0.03	0.15±0.06
	σ_e^2	1.12±0.05	0.99±0.06	0.90±0.06
	m^2	0.00±0.00	0.03±0.03	0.12±0.04
MINB	σ_{pen}^2	0.14±0.03	0.15±0.02	0.14±0.03
	σ_m^2	3.8E-06±0.00	0.007±0.01	0.08±0.03
	σ_e^2	0.54±0.05	0.53±0.03	0.47±0.03
	m^2	0.00±0.00	0.01±0.02	0.11±0.04
MINL	σ_{pen}^2	6.53±1.12	6.93±1.23	6.66±1.21
	σ_m^2	2.4E-06±0.00	0.18±0.67	2.70±1.45
	σ_e^2	23.4±1.17	25.76±1.42	23.66±1.63
	m^2	0.00±0.00	0.005±0.02	0.08±0.04
PH	σ_{pen}^2	0.012±0.002	0.013±0.002	0.013±0.002
	σ_m^2	1.5E-09±0.00	0.00016±0.0007	0.002±0.001
	σ_e^2	0.033±0.001	0.033±0.002	0.031±0.002
	m^2	0.00±0.00	0.003±0.01	0.04±0.03
SCOL	σ_{pen}^2	0.03±0.01	0.03±0.01	0.03±0.00
	σ_m^2	1.2E-09±0.00	1.8E-07±0.00	0.03±0.01
	σ_e^2	0.29±0.01	0.29±0.01	0.280.01
	m^2	0.00±0.00	0.00±0.00	0.06±0.04
SFIRM	σ_{pen}^2	0.05±0.03	0.05±0.03	0.04±0.03
	σ_m^2	4.2E-07±0.00	5.8E-07±0.04	0.14±0.05
	σ_e^2	1.00±0.05	0.97±0.05	0.88±0.05
	m^2	0.00±0.00	0.00±0.00	0.14±0.04
SSF	σ_{pen}^2	1.34±0.42	1.28±0.41	1.29±0.42
	σ_m^2	0.23±0.36	1.63±0.64	0.38±0.05
	σ_e^2	11.55±0.68	10.40±0.71	11.43±0.67
	m^2	0.01±0.02	0.12±0.05	0.03±0.03

[2]LD = Loin depth; FD = Fat depth; CADG = Carcass average daily gain; IMF = Intramuscular fat percent, MINA = Minolta a*, MINB = Minolta b*, MINL = Minolta L*, PH = Ultimate pH; SCOL = Subjective color score; SMARB = Subjective marbling score; SFIRM = Subjective firmness score; SSF = Slice shear force, HAM = Ham weight; LOIN = Loin weight; BEL = Belly weight

Table S7: Spearman correlation among genomic estimated breeding values (gEBVs) estimated with the different models.

Trait	M0.vs.M1	M0.vs.M2	M0.vs.M3
LD	0.998	0.976	0.963
FD	0.997	0.984	0.973
CADG	1	0.986	0.97
HamWt	1	0.987	0.986
LoinWt	0.999	0.998	0.997
BellyWt	1	0.999	1
IMF	0.999	0.992	0.99
MinA	1	1	0.994
MinB	1	1	0.989
MinL	1	1	0.999
pH	1	1	0.998
Scol	1	1	0.998
Smarb	1	0.987	0.989
Sfirm	1	0.996	1
SSF	0.999	0.996	0.999
**Average**	**0.999**	**0.993**	**0.990**

Model 0 contains genomic and pen effect as random effect, Model 1, Model 2 and Model 3 contains microbial effect at weaning, Mid-test and Off-test in addition to genomic and pen effect.

Table S8: Spearman correlation between genomic estimated breeding values (gEBVs) and estimated microbial values (EMVs) for the three different models

**Trait**	**M1**	**M2**	**M3**
LD	0.286	0.328	0.355
FD	0.298	0.344	0.351
CADG	0.279	0.305	0.405
HamWt	0.290	0.281	0.297
LoinWt	0.302	0.315	0.392
BellyWt	0.276	0.318	0.390
IMF	0.311	0.331	0.299
MinA	0.172	0.284	0.376
MinB	0.174	0.266	0.304
MinL	0.248	0.302	0.393
pH	0.245	0.162	0.323
Scol	0.239	0.289	0.293
Smarb	0.310	0.269	0.358
Sfirm	0.287	0.365	0.342
SSF	0.225	0.336	0.248
**Average**	**0.263**	**0.300**	**0.342**

Model 0 contains genomic and pen effect as random effect, Model 1, Model 2 and Model 3 contains microbial effect at weaning, Mid-test and Off-test in addition to genomic and pen effect.

Table S9. Estimates of genomic correlation (below diagonal) at among meat quality traits.

	[1]**SCOL**	**IMF**	**SFIRM**	**MINA**	**MINB**	**PH**
**SCOL**						
**IMF**	-0.24±0.13					
**SFIRM**	0.16±0.19	[2]**0.36±0.15**				
**MINA**	**0.45±0.16**	**0.29±0.14**	-0.38±0.26			
**MINB**	**-0.94±0.22**	**0.78±0.16**	-0.06±0.31	-0.02±010		
**PH**	**0.91±0.29**	-0.18±0.25	0.42±0.35	-0.05±0.31	-0.53±0.42	

Table S10. Estimates of genomic correlation (below diagonal) at among carcass composition traits.

	[1]FD	CADG	HAM	LOIN	BEL
**FD**					
**CADG**	**0.27±0.13**				
**HAM**	0.03±0.17	**0.67±0.13**			
**LOIN**	-0.11±0.15	**0.69±0.10**	**0.54±0.19**		
**BEL**	**0.62±0.11**	**0.79±0.07**	**0.58±0.19**	**0.70±0.03**	

Table S11. Estimates of genomic correlation between meat quality traits and carcass composition traits with inclusion of microbial effect

	[1]FD	CADG	HAM	LOIN	BEL
**SCOL**	0.06±0.13	-0.10±0.17	-0.11±0.19	-0.04±0.16	-0.07±0.17
**IMF**	**0.23±0.11**	0.08±0.15	-0.05±0.16	-0.03±0.14	**0.28±0.14**
**SFIRM**	**0.27±0.13**	0.09±0.23	0.09±0.25	0.10±0.22	**0.46±0.23**
**MINA**	**0.27±0.13**	-0.10±0.21	-0.31±0.22	-0.24±0.21	-0.15±0.21
**MINB**	**0.42±0.21**	0.19±0.26	0.02±0.20	0.08±0.26	0.13±0.27
**PH**	-0.02±0.26	0.10±0.32	-0.19±0.28	0.09±0.20	-0.30±0.33

Table S12. Estimates of genomic correlation between meat quality traits and carcass composition traits without inclusion of microbial effect

	[1]FD	CADG	HAM	LOIN	BEL
**SCOL**	-0.06±013	-0.01±0.17	-0.05±0.18	-0.02±0.17	-0.10±0.17
**IMF**	**0.26±0.10**	0.13±0.14	0.01±0.16	-0.02±0.14	**0.37±0.14**
**SFIRM**	**0.28±0.08**	0.13±0.28	0.12±0.24	0.02±0.22	**0.51±0.18**
**MINA**	**0.30±0.14**	0.09±0.17	-0.27±0.21	-0.28±0.20	-0.05±0.21
**MINB**	**0.46±0.19**	0.14±0.27	0.02±0.25	-0.02±0.27	0.16±0.27
**PH**	0.01±0.24	0.12±0.31	-0.28±0.36	0.06±0.31	-0.26±0.32

[1]FD = Fat depth; CADG = Carcass average daily gain; IMF = Intramuscular fat percent, MINA = Minolta a*, MINB = Minolta b*, MINL = Minolta L*, PH = Ultimate pH; SCOL = Subjective color score; SMARB = Subjective marbling score; SFIRM = Subjective firmness score; HAM = Ham weight; LOIN = Loin weight; BEL = Belly weight;

[2]Numbers in bold are significant.
